# Association of Symptoms of Attention-Deficit/Hyperactivity Disorder with Physical Activity, Media Time, and Food Intake in Children and Adolescents

**DOI:** 10.1371/journal.pone.0049781

**Published:** 2012-11-14

**Authors:** Andreas W. A. van Egmond-Fröhlich, Daniel Weghuber, Martina de Zwaan

**Affiliations:** 1 Department of Pediatrics, SMZ-Ost Donauspital, Vienna, Austria; 2 Department of Pediatrics, Paracelsus Private Medical School, Salzburg, Austria; 3 Department of Psychosomatic Medicine and Psychotherapy, Hannover Medical School, Germany; University of Texas Southwestern Medical Center, United States of America

## Abstract

**Introduction:**

The aim of the study was to assess the association between attention deficit/hyperactivity disorder (ADHD) symptoms and potentially obesogenic behaviors.

**Methods:**

Data of 11,676 German children and adolescents (6–17 years) were analyzed. Television/video exposure, physical activity, food frequency and portion size were assessed using questionnaires. A dietary quality index, energy density and volumes of consumed food, and total energy intake were calculated. The parent-rated hyperactivity/inattention subscale of the Strengths and Difficulties Questionnaire (SDQ-HI) was used as a continuous measure of ADHD symptoms. Associations were analyzed with general linear models adjusting for sex, age, socioeconomic status, migrant status, parental BMI, and parental smoking.

**Results:**

SDQ-HI scores correlated positively with physical activity, average energy density of food, volume of beverages, total energy intake, and television exposure and negatively with the nutritional quality score (HuSKY) even after adjustment for parental variables (BMI, smoking, socioeconomic status, migrant status), age, sex, as well as the other SDQ subscales. The adjusted association of the SDQ-HI scores with the nutritional quality score was stronger in girls and the associations with food volume, food energy, and total energy intake was significant only in girls.

**Conclusions:**

Poor nutritional quality, high energy intake and television exposure appear to be independently associated with ADHD symptoms. The relationship between food energy intake and ADHD symptoms was especially pronounced in girls and this may help to explain the reported association of ADHD symptoms with overweight in adolescent girls.

## Introduction

Attention-deficit/hyperactivity disorder (ADHD) is characterized by inattention, hyperactivity, and impulsivity [1] and is the most prevalent mental health disorder in childhood. While there are many clinical and epidemiological publications on the association between ADHD or ADHD symptoms and overweight/obesity [2], [3], [4], [5], [6], [7], [8], [9], [10], [11], [12], [13] the literature on the association between ADHD symptoms and energy balance related behaviors such as physical activity, television exposure, and food and beverage intake is scarce.

### ADHD Symptoms and Physical Activity

Motor hyperactivity, especially regarding size and variability of movements, seems to be a core feature of both the combined type and the hyperactive-impulsive type of ADHD [14]. Children with ADHD show increased physical activity both during the night [15], [16] and especially during structured school activities [15]. On the other hand children and adolescents with ADHD appear to be less likely to engage in regular vigorous physical activity and organized sports [17].

### ADHD Symptoms and Television Exposure

Television exposure may be associated with childhood obesity for several reasons. First it is a sedentary activity that reduces time spent in more active enterprises [17]. Second, television exposure results in the viewing of advertisements for energy dense foods [18], [19], [20]. Third, while viewing television, children eat more energy dense snacks [21]. Most cross-sectional studies have found attention problems [22], [23] and ADHD symptoms [24] to be associated with more media time. With the exception of one study that lacked an acceptable measure of ADHD [25] all longitudinal studies reported evidence that television exposure is a risk factor for subsequent attention problems [24], [26], [27], [28].

### ADHD and Choice of Food

It has been suggested, though not empirically proven, that impulsive children tend to overeat in an obesogenic environment [29], [30], [31]. In adults low inhibitory control was associated with unintentional overeating in a variety of experimental studies [32], [33], [34], [35] regardless of weight status.

In one cross-sectional study in 375 school-aged children, ADHD was significantly associated with increased intake of sweets and fast-food measured by a semi-quantitative food-frequency questionnaire [36]. Factor analysis of dietary data revealed that adolescents with ADHD were likely to consume a “Western” diet high in saturated and total fat and refined sugar while low in fiber, but unlikely to consume a Mediterranean diet rich in fish, vegetables, fruit, legumes, and whole-grain foods after adjusting for known confounding factors [37].

To our knowledge no study has investigated the association between ADHD or ADHD symptoms and relevant behavioral parameters such as quality of nutrition, energy density of foods, or energy intake simultaneously while carefully controlling for putative confounding variables. It has been shown that parent-rated hyperactive behavior (SDQ-HI) [38], television exposure [39], and poor food choices [40] are all significantly and negatively associated with socioeconomic status. A recent study by van Egmond-Fröhlich et al. [13] demonstrated that the association between ADHD symptoms and overweight/obesity is not independent of confounding factors such as parental BMI, parental smoking, and parental socioeconomic status. Only in the subgroup of adolescent girls ADHD symptoms were independently associated with overweight/obesity. This was confirmed in a study involving the National Survey of Children’s Health in the US [41]. The authors conclude that obesity per se may not have a direct association with ADHD symptoms.

The cross-sectional German Health Interview and Examination Survey for Children and Adolescents (KiGGS) provides comprehensive and nationally representative data of children living in Germany. The KiGGS study allows the examination of the association between ADHD symptoms based on the parent-rated SDQ-hyperactivity-inattention subscale (SDQ-HI) and behaviors relevant for obesity such as television exposure, physical activity, energy density of food, and energy intake, while adjusting for a variety of possible confounding variables. A more detailed knowledge of the association and interaction between health behaviors, gender, individual risk factors (e.g. ADHD symptoms), and parental risk factors is required to design effective preventive and therapeutic interventions in childhood.

Based on previous results we expected that the SDQ-HI subscale score would be positively correlated with high television exposure, high energy density and energy intake, and negatively correlated with high dietary quality. According to our earlier findings [13], we also expected to find an attenuation of the association between the SDQ-HI scores, dietary quality, and energy intake after adjusting for confounding parental variables. Finally we expected a significant interaction between SDQ-HI scores and gender (with stronger effects in girls) regarding energy intake.

## Methods

### Sample

From May 2003 to May 2006, a total of 17,641 children and adolescents aged 0–17 years participated in the cross-sectional population survey KiGGS. The sample was derived from 167 representative German communities, as described elsewhere [42].

Information about sociodemographic characteristics, living conditions, health, and health behaviors were obtained with self-administered questionnaires filled in by the parents [43]. The survey was approved by the Federal Office for Data Protection and by the ethics committee of the Medical School of the Charité-University Berlin.

The present analysis is based on children and adolescents aged 6–17 years (n = 11,967). After excluding 291 subjects with missing data on the parent-rated SDQ-HI subscale and missing data regarding socioeconomic status, migrant status, parental smoking, and parental BMI, the number of participants was reduced to n = 9,428 (these subject numbers were determined before weighting).

### Data Obtained from Parental Questionnaires for All Ages

The parent-rated *Strengths and Difficulties Questionnaire* (SDQ) assesses mental health problems in children and adolescents aged 4–16 years. Using clinical interviews as a gold standard it outperformed the Child Behavior Checklist at detecting inattention and hyperactivity [44]. Because of its reliability and validity [45], [46] the SDQ is considered a useful brief measure. The five subscales, emotional symptoms, conduct problems, hyperactivity/inattention (HI), peer problems, and pro-social behavior, consist of 5 items each. The 5 items of the HI subscale capture the three key symptom domains relevant for ADHD symptomatology: inattention (2 items: “easily distracted, concentration wanders”; “good attention span, sees chores or homework through to the end”), hyperactivity (2 items: “restless, overactive, cannot stay still for long”; “constantly fidgeting or squirming”), and impulsivity (1 item: “thinks things out before acting”).

The items were rated by the parents as “not correct”, “partly correct”, or “completely correct”. Each subscale has possible values from 0 to 10. Cronbach’s alphas in our sample were .663 (emotional symptoms), .548 (conduct problems), .775 (hyperactivity/inattention), .603 (peer relationship problems), and .652 (pro-social behavior). We adopted the cut-offs for borderline and abnormal scores of the SDQ-HI subscale from the English normative data [47].

We used this instrument as a continuous measure of ADHD symptoms rather than a categorical measure for ADHD diagnosis since a large-scale twin study [48] and a sibling study [49] have provided strong evidence that ADHD may be better viewed as the extreme end of a continuum rather than as a category.

### Socioeconomic Status, Migrant Status, Parental Body Mass Index, Parental Smoking

The socioeconomic status score was calculated from parental occupational status and education as well as family income [50], [51]. Migrant status was defined as at least two out of mother, father, and child being born outside Germany [43]. Parental BMI was calculated from self-reported height and weight. Only the weights and heights of those parents were used who identified themselves as the biological parents. Parental smoking at the time of assessment was reported in the questionnaire using the categories “never”, “occasionally”, “regularly”.

### Television/Video Exposure

In 6 to 13 year olds parents reported their children’s approximate average daily hours of television and video viewing separate for weekdays and weekends. Response options were “none”, “30 minutes”, “1–2 hours”, “3–4 hours”, and “more than 4 hours” which were recoded (using 0, 0.5, 1.5, 3.5, and 5, respectively) into average hours of use per day, then weighted with 5/7 for weekday exposure and with 2/7 for weekend exposure, and summed up. Children and adolescents aged 11 years and older were asked directly about their daily media time with identical response options (but not differentiating weekday and weekend exposure). The responses were recoded into average hours of use per day as above. In 11–13 year old participants both parent-ratings and self-ratings were available. Hence, in this age group both ratings were combined and means were calculated.

### Physical Activity

Data on physical activity are only available in children and adolescents aged 11 years and older by self-report. They were asked to report the hours per week of leisure time physical activity (e.g. organized sports, bicycling) where they really start to sweat or get out of breath. This was used as a measure of weekly hours of medium to high intensity physical activity.

### Dietary Assessment

Parents of children under the age of 11 and children and adolescents aged 11 and older filled in the semi-quantitative food frequency questionnaire (FFQ) [52]. Frequency and portion size of 9 beverages and 36 food items were assessed asking about their consumption in the “last few weeks”.

The responses regarding frequencies and portion sizes were calculated into average portions per day and food weights or beverage volumes per portion of each item. Frequencies and portion sizes were then multiplied to estimate the daily amount consumed of each item.

The average energy density values for each item of the FFQ was derived from a subgroup of 12–17 year old participants in the KiGGS survey, who also contributed detailed dietary assessments as participants in the EsKiMo (Eating Study as a KiGGS Module) [53] using DISHES (Dietary Interview Software for Health Examination Studies). The average energy densities of food (in kJ/100 g) and beverages (in kJ/100 ml) were calculated as the average energy densities of the corresponding items in the FFQ weighted by the individual average daily consumption of the items.

The daily energy intake of food and of beverages as well as the total daily energy intake (in kJ/d) was estimated as the sum of the daily energy intakes from the items. This daily energy intake was calculated for each item as the product of energy density and the average daily amount consumed.

As an alternative analysis we also calculated the Healthy Nutrition Score for Kids and Youth (HuSKY) based on the FFQ items as described in detail elsewhere [40]. Briefly we first calculated for each of the eleven food groups (beverages, fruit, vegetables, bread/cereals, pasta/rice/potatoes, milk/dairy products, eggs, meat/sausage, fish, butter/margarine, sweets/snacks/sugared drinks) the ratio between reported food intake and the age- and sex-specific food-based dietary recommendation for children and adolescents [54]. The food ratios were then recoded into points ranging from 0 to 100 for every food group. The maximum of 100 points was given when the recommendation for the food group was met. For food groups with a recommendation to consume plenty, lower amounts received linearly less points while higher consumptions were assigned 100 points. For food groups with the recommendation to consume small amounts, consumptions at or below the recommendation were assigned 100 points and higher amounts were assigned descending points reaching zero at double the recommended consumption. For food groups, for which moderate consumption is recommended, points were assigned by an inverted V-shaped function. The resulting 11 subscores were added and standardized to a scale ranging from zero to 100. A higher score corresponds to a better dietary quality.

### Statistical Analyses

The health behavior variables included television exposure, days with moderate to vigorous physical activity, as well as energy intake and the HUSKY nutritional quality score. In order to identify putative differential associations between ADHD symptoms and different aspects of food and beverage intake, we examined volume and energy density of food and beverages separately in addition to total energy intake.

We selected potential parental confounders based on their association with both the SDQ-HI scores and overweight status as demonstrated in a previous study using the same database [13]. In order to verify whether these parental variables and migrant status are confounders that require adjustment, we first conducted general linear models (GLM) with SDQ-HI as the dependent variable and the potential parental confounders (parental BMI, parental smoking, socioeconomic status, migrant status) as independent variables adjusting for age and sex. Secondly we examined whether the potential confounders were independently associated with the health behavior variables after adjusting for the other potential confounders, age, sex, and SDQ-HI.

We used separate GLMs to examine the association of SDQ-HI scores with each of the health behavior variables. SDQ-HI scores were entered as the independent variable in each GLM. In a first model we adjusted only for age and sex and in a second model also for the parental confounding variables (shown to be associated with both the SDQ-HI scores and the behavioral variables) and the other SDQ subscales.

We also examined interactions of the SDQ-HI scores with sex and age regarding the health behavior variables.

As a sensitivity analysis we eliminated subjects for whom the parents reported medication for neurological conditions including medication for ADHD.

We used a weighing procedure [42] for age, sex, state of residence (Western or Eastern Germany), and nationality according to the distribution of these socio-demographic factors in the German population as given by the Federal Statistics Office.

A p-value <0.05 (in interaction analyses p<0.10) was considered to be statistically significant. All analyses were performed using SPSS (version 17.0 for Windows; SPSS, Inc., Chicago, IL).

## Results

### Description

The descriptions of the health behavior variables are listed in Table 1.

**Table 1:** Description of the health behaviors.

Variable	N§	Mean	SD	P25	Median	P75
Television/video exposure (h/d)	12060	1.6	1.1	0.8	1.5	1.8
Medium-high intensity physical activity (h/wk)$	6300	6.9	7.1	3	5	9
HuSKY nutritional quality score (0–100)	9462	55	11	48	55	62
Food energy density (kJ/100 g)	11553	955	136	868	953	1042
Beverage energy density (kJ/100 ml)	11553	100	58	51	100	148
Food volume (g/d)	11553	1094	593	727	966	1292
Beverage volume (ml/d)	11553	1922	1494	919	1439	2531
Food energy intake (MJ/d)	11553	10.52	6.26	6.72	9.04	12.38
Beverage energy intake (MJ/d)	11553	1.74	1.71	0.63	1.20	2.16
Total energy intake (MJ/d)	11520	12.2	6.8	8.0	10.6	14.4

§ N is calculated after weighting and therefore exceeds the number of participants.

$ only available n 11–16 years old participants.

P25 = 25^th^ percentile; P75 = 75^th^ percentile.

The mean (SD) SDQ-HI subscale score was 3.1±2.3; it was normal in 86.8%, borderline in 5.7%, and abnormal in 7.8% of the participants.

Social class was low in 27%, medium in 46%, and high in 27% of the children and adolescents. Daily smoking was reported by 25.2% of the mothers and 31.8% of the fathers where data were available. Of the mothers and fathers 24.2% and 47.4% were overweight but not obese and 12.5% and 13.5% were obese, respectively. Employing International Obesity Task Force (IOTF) methods and using German BMI percentiles [55] 5.2% of the children were obese, 15.1% overweight but not obese, and 6.7% underweight.

### Association of Parental Variables and Migrant Status with SDQ-HI Scores and Health Behavior Variables

Maternal (p<0.0005) and paternal (p<0.005) BMI, maternal (p<0.0005) and paternal (p<0.0005) smoking, as well as migrant status (p<0.05) were significantly and positively associated with the SDQ-HI score whereas socioeconomic status (p<0.0005) was negatively associated with the SDQ-HI score after adjusting for the other potential confounders, age, and sex.

The associations between the parental variables (BMI, smoking, socioeconomic status, migrant status) and the health behaviors are summarized in Table 2. The directions of the associations were in line with our expectations. As all examined parental variables were significantly associated with at least one behavioral variable we decided to adjust for all of them.

**Table 2:** Associations of parental variables (BMI, smoking, SES, migrant status) with health behaviors adjusted for SDQ-HI, age, and sex.

Health behavior variables asdependent variables	N§	MaternalBMI	PaternalBMI	Maternalsmoking	Paternalsmoking	Socioeconomicstatus	Migrant
Television/video exposure (h/d)	9.199	.004***	.002***	.005***	.002***	.030***	.006***
Medium-high intensity physical activity (h/wk)$	4.863	n.s.	n.s.	n.s.	n.s.	.*002* *	n.s.
HuSKY nutritional quality score	7.325	n.s.	.001**	.*001* *	n.s.	.013***	n.s.
Food energy density	8.857	n.s.	n.s.	n.s.	n.s.	.005***	.021***
Beverage energy density	8.856	.002***	n.s.	.001**	n.s.	.006***	.005***
Food volume	8.857	n.s.	n.s.	n.s.	n.s.	.001**	.011***
Beverage volume	8.856	n.s.	.001**	.003**	.001**	.005***	n.s.
Food energy intake	8.857	n.s.	n.s.	n.s.	n.s.	.002***	.019***
Beverage energy intake	8.895	n.s.	n.s.	.004***	.002***	.009***	.003***
Total energy intake	8.839	n.s.	n.s.	n.s.	.001**	.005***	.013***

All parental variables were entered together. Partial eta^2^ values for significant independent associations are listed (with negative associations in italic style).

§ N is calculated after weighting and therefore exceeds the number of participants.

* P<0.05; **p<0.01; ***p<0.001.

$ only available n 11–16 years old participants.

### Association of Health Behavior Variables with SDQ-HI Scores

In the models adjusted for sex and age only (Table 3) SDQ-HI scores were significantly and positively associated with television exposure, medium to high intensity physical activity (only available in 11–17 year old participants), and total energy intake, while they were negatively associated with the HuSKY diet quality index.

**Table 3:** Associations of SDQ-HI scores with health behaviors adjusted for age and sex only.

Health behavior variables asdependent variables		SDQ-HI as independent variable			
	N§	B	95% CI	P	partial eta^2^
Television/video exposure (h/d)	11 349	0.064	0.055–0.073	0.000	0.017
Medium-high intensity physical activity (h/wk)$	6 098	0.227	0.143–0.310	0.000	0.005
HuSKY nutritional quality score (0–100)	8 905	−0.565	−0.661 – −0.468	0,000	0,015
Food energy density (kJ/100 g)	10 861	5.60	4.42–6.77	0.000	0.008
Beverage energy density (kJ/100 ml)	10 864	1.06	0.56–1.54	0.000	0.002
Food volume (g/d)	10 861	6.1	1.0–11.2	0.020	0.001
Beverage volume (ml/d)	10 864	48.1	35.6–61.2	0.000	0.005
Food energy intake (kJ/d)	10 861	120	66–174	0.000	0.002
Beverage energy intake (kJ/d)	10 915	61	47–76	0.000	0.006
Total energy intake (kJ/d)	10 830	181	123–240	0.000	0.003

§ N is calculated after weighting and therefore exceeds the number of participants.

$ only available n 11–16 years old participants.

Weakened but mostly still significant relationships between SDQ-HI scores and health behaviors were found in the models adjusting for parental variables (BMI, SES, smoking, and migrant status) as well as age and sex (not shown).

When we additionally adjusted for the other 4 SDQ subscales (conduct problems, emotional problems, peer problems, pro-social behavior) the associations between the SDQ-HI subscale score and the behavioral variables remained statistically significant with the exception of energy intake from food. However, regression coefficients were moderately reduced (Table 4).

**Table 4:** Associations of SDQ-HI scores with health behaviors adjusted for the potential parental confounders (migrant status, parental BMI, SES, and smoking), age, sex, as well as the other SDQ-subscales.

Health behavior variables asdependent variables		SDQ-HI as independent variable			
	N§	B	95% CI	P	partial eta^2^
Television/video exposure (h/d)	9 199	0.021	0.010–0.032	0.000	0.002
Medium-high intensity physical activity (h/wk)$	4 863	0.056	0.014–0.232	0.026	0.001
HuSKY nutritional quality score (0–100)	7 325	−0.365	−0.478 – −0.252	0.000	0,004
Food energy density (kJ/100 g)	8 857	4.18	2.68–5.68	0.000	0.003
Beverage energy density (kJ/100 ml)	8 856	0.98	0.35–1.62	0.002	0.001
Food volume (g/d)	8 857	0.5	−6.0–7.0	0.881	0.000
Beverage volume (ml/d)	8 856	16.6	0.7–32.6	0.041	0.000
Food energy intake (kJ/d)	8 857	44	−25–112	0.209	0.000
Beverage energy intake (kJ/d)	8 895	36	18–54	0.000	0.002
Total energy intake (kJ/d)	8 839	80	5–154	0.035	0.000

§ N is calculated after weighting and therefore exceeds the number of participants.

$ only available in 11–16 years old participants only.

### Interactions of the SDQ-HI Scores with Sex and Age Regarding Health Behavior Variables

Significant interactions of SDQ-HI scores with sex were found for the dietary quality index, food volume and energy intake from food, and as a trend also for total energy intake (Table 5). The association of the SDQ-HI scores and the dietary quality index was stronger for girls than for boys and for the other above mentioned behavioral variables it was significant only in girls but not in boys.

**Table 5:** Associations of SDQ-HI scores with health behaviors: Interactions with sex.

Health behavior variables asdependent variables		SDQ-HI x sex interaction as independent variable per unit SDQ-HIfor girls (with boys as reference)			
	N§	B	95% CI	P	partial eta^2^
Television/video exposure (h/d)	9 199	−0.003	−0.021 – 0.016	0.791	0.000
Medium-high intensity physical activity (h/wk)$	4 863	−0.089	−0.277 – 0.099	0.352	0.000
HuSKY nutritional quality score (0–100)	7 325	−0.110	−0.485 – −0.054	0,014	0,001
Food energy density (kJ/100 g)	´8 857	0.334	−2.27–2.94	0.802	0.000
Beverage energy density (kJ/100 ml)	8 856	0.60	−1.05–1.17	0.916	0.000
Food volume (g/d)	8 857	14.2	3.0–25.5	0.013	0.001
Beverage volume (ml/d)	8 856	−13.2	−40.8–14.4	0.348	0.000
Food energy intake (kJ/d)	8 857	143	25–262	0.018	0.001
Beverage energy intake (kJ/d)	8 895	−20	−51–21	0.226	0.000
Total energy intake (kJ/d)	8 839	124	−5–53	0.060	0.000

GLM adjusted for SDQ-HI, migrant status, parental BMI, SES, and smoking as well as age and sex.

§ N is calculated after weighting and therefore exceeds the number of participants.

$ only available in 11–16 years old participants.

There was no significant interaction of SDQ-HI scores with age regarding any of the behavioral variables for either or both sexes.

All results were virtually unchanged when participants taking medication for neurological conditions (e.g. stimulant medication for ADHD or antiepileptic medication known to affect food intake) were omitted from the analyses.

## Discussion

As expected from the previous research mentioned in the introduction we found significant associations between ADHD symptoms, low dietary quality, and high total energy intake. We could demonstrate that these associations were not entirely explained by the investigated parental variables or other psychopathology of the children. The association with the SDQ-HI scores was more pronounced for food quality than for food volume. In detail, the significant positive association between SDQ-HI scores and energy intake from beverages was due to both increased volume and increased energy density of the beverages. The association between SDQ-HI scores and energy intake from food was explained by the energy density of food but not by food volume. This might indicate that ADHD symptoms may be associated with poor food selection rather than overeating in terms of volume. This is in line with the results of a recent cross-sectional study showing that Australian children with ADHD symptoms were more likely to consume a “Western” diet high in saturated and total fat and refined sugar while low in fiber, than to consume a Mediterranean diet rich in fish, vegetables, fruit, legumes, and whole-grain foods [37].

We found that girls showed a significantly stronger association between ADHD symptoms and dietary quality, food volume, food energy intake, and (marginally) total energy intake than boys. This corresponds with our previous results based on the same population [13], where we found a significant independent association between ADHD symptoms and overweight only in adolescent girls. The success of treatment for obesity is reduced by ADHD symptoms or impulsivity in most studies [29], [56], [57]. Girls are under stronger societal pressure to reduce weight and food intake than boys. In those motivated to do so, the success in reducing intake of high energy food may depend on attention and impulse control and thus be inversely related to ADHD symptoms.

We could confirm the independent association between ADHD symptoms and television and video exposure [22], [23], [24]. This is in line with the recently published results of a cross-sectional study of 68,634 children from the National Survey of Children’s Health in the United States [41]. The authors reported that an average TV usage during weekdays of 1 hour or more was significantly associated with a diagnosis of ADHD even after adjusting for very similar parental variables as used in our study. The design of our study does not allow causative interpretations, but there is growing evidence in the literature that exposure to television and video games in childhood may actually be associated with increased subsequent attention problems [24].

The weak but significant positive association between ADHD symptoms and medium to high intensity physical exercise in the 11–17 year old participants is in line with studies using actometry [14], [15], [16] but contradicts the results of large US population based studies which found that children with ADHD symptoms were less likely to participate in vigorous physical activity and organized sports [17], [41]. However, we did not assess participation in organized sports and clubs. The negative association between participation in organized sports and ADHD symptoms found in the US studies could be due to the fact that children and adolescents with ADHD symptoms might have difficulties in sports teams because of their behavioral problems. In addition, different age ranges and predictor variables (ADHD symptoms vs. ADHD diagnosis) as well as possible cultural differences might also explain this discrepancy.

Parental variables such as socioeconomic status, BMI, and smoking have been shown to be strongly associated with ADHD symptoms, television exposure, and dietary quality. However, these confounding variables did not entirely explain the positive association between ADHD symptoms and television exposure, dietary quality, and energy intake in our sample. In addition, the associations between the SDQ-HI subscale scores and the health behaviors remained significant for all but food energy intake when adjusting for the other SDQ-subscales and are, thus, largely independent of peer relations, emotional problems and other behavioral problems assessed with the SDQ.

### Strengths and Limitations

The main strengths of our report lie in the large representative study on which it is based, and the multivariate analyses that allowed looking for independent effects of ADHD symptoms. ADHD symptoms were used as a dimensional and not a categorical variable. In our effort to control for parental confounding variables, we included a sophisticated socioeconomic status score, migrant status, and parental smoking in addition to parental BMI, age, and sex.

However, given the cross-sectional nature of the data, we cannot establish the direction of the detected relationships. In addition, the health behavior variables used in our study were based on self-reported data and are thus liable to social desirability and recall bias. Also, we have combined parent-rated and self-rated television exposure time because only the combined variable spans the whole age range of 6–17 years used in our study. Fortunately, both ratings were significantly associated with SDQ-HI scores, also after adjusting for parental confounders as well as sex and age.

Finally, while the associations were statistically significant, they were generally weak, explaining less than 1% (derived from the partial eta^2^ values given in Table 4 multiplied by 100%) of the variance of each of the variables adjusted for age, sex, and parental confounders. The imprecision of measuring behavior by self-report and parental report may have mitigated the observed associations.

### Conclusions

There is evidence that overweight/obese children have a significantly higher risk for ADHD symptoms than normal weight children. Our study adds to this finding in that poor nutrition and high television exposure time also seem to directly be associated with ADHD symptoms even after adjusting for potential confounding variables. Clinicians should be aware that children and adolescents with ADHD symptoms should be monitored with regard to food intake and television/video exposure. Environmental control measures and parental monitoring may be required to improve the dietary patterns and to reduce TV usage time of children and adolescents with ADHD symptoms, as previous studies have shown that these individuals profit less from behavioral weight loss interventions [57], [58]. Also preventive efforts to promote healthy behaviors will have to address the network of associations between ADHD symptoms, health behaviors, overweight/obesity, and parental variables.

Finally, further research based on large longitudinal cohorts is needed to address the direction of causality.
